# *In Vitro* and *in Vivo* Antitumor Activity of Scutebarbatine A on Human Lung Carcinoma A549 Cell Lines

**DOI:** 10.3390/molecules19078740

**Published:** 2014-06-25

**Authors:** Xiao-Kun Yang, Ming-Yuan Xu, Gui-Sen Xu, Yu-Lan Zhang, Zhao-Xia Xu

**Affiliations:** Department of Emergency, Chengdu Military General Hospital, Chengdu 610083, China; E-Mails: mingyuanxucz@yeah.net (M.-Y.X.); guisenxucdzy@126.com (G.-S.X.); ylzhangcz@126.com (Y.-L.Z.); xuzaox2014@163.com (Z.-X.X.)

**Keywords:** scutebarbatine A, antitumor, apoptosis, A549 cells, lung cancer

## Abstract

During our systematic study on the anticancer activities of *Scutellaria barbata*, scutebarbatine A (SBT-A), one of the major alkaloids in *S. barbata*, was found to have antitumor effects on A549 cells. Thus, we designed the present study to investigate in detail the antitumor effects of SBT-A. The cytotoxic effect of SBT-A on A549 *in vitro* were determined by an MTT assay and evaluated by IC_50_ values. Furthermore, results of Hoechst 33258 and Annexin V/PI staining assays demonstrated that SBT-A had significant antitumor effects on A549 cells via apoptosis, in a concentration-dependent manner. What’s more, the mechanism was explored by western blotting, and our study revealed that SBT-A can up-regulate the expressions of cytochrome c, caspase-3 and 9, and down-regulate the levels of Bcl-2 in A549 cells. Finally, the antitumor effects of SBT-A were evaluated *in vivo* by using transplanted tumor nude mice, and the results confirmed that SBT-A has a notable antitumor effect on A549 cancer via mitochondria-mediated apoptosis. Collectively, our results demonstrated that SBT-A showed significant antitumor effects on A549 cells *in vivo* and *in vitro* via mitochondria-mediated apoptosis by up-regulating expressions of caspase-3 and 9, and down-regulating Bcl-2.

## 1. Introduction

Lung cancer is the leading cause of cancer-related death in both men and women worldwide [1,2]. Lung cancers are commonly classified as small cell lung cancer (SCLC) and non-small cell lung cancer (NSCLC), among which NSCLC constitutes approximately 75% of lung cancer cases nowadays [3]. Improvement have been made in diagnosis and treatment of lung cancers, however it remains an aggressive cancer with a poor prognosis [4]. Traditional Chinese Medicines are folk medicines that have been used in China for thousands of years; in recent years, more and more researchers have recognized that the TCMs are the potential source of novel and effective anticancer drugs [5,6] and considerable numbers of agents with promising anticancer activities have been isolated from TCMs [7,8].

**Figure 1:**
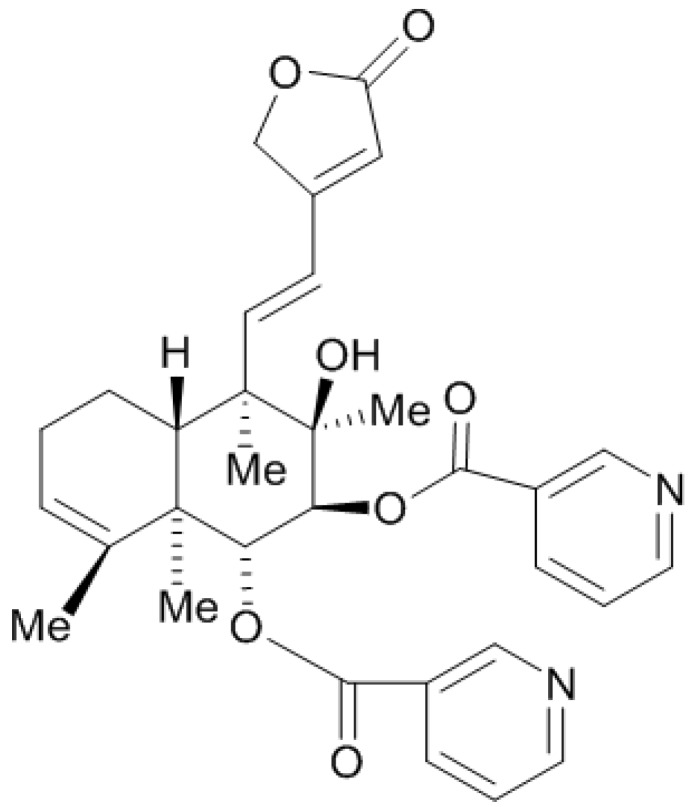
The chemical structure of scutebarbatine A.

*Scutellaria barbata* D. Don, a perennial herb belonging to the family Lamiaceae, is widely distributed throughout China and Korea. It has been traditionally used in folk medicine as an antiinflammatory and antitumor agent [9,10,11,12]. *S. barbata* is known to contain a large number of alkaloids, flavones, polysaccharides, organic acids, and neoclerodane diterpenoids [13,14,15,16]. In our previous research, we found that the polysaccharides of *S. barbata* had significant inhibitory effect on invasion and metastasis of 95-D cell lines [17]. As a part of our systematic study on the anticancer activities of *S. barbata*, we further found in preliminary experiments that scutebarbatine A (SBT-A, Figure 1), which is one of the major alkaloids in *S. barbata*, showed a notable anticancer effects on the A549 cell line (a classical NSCLC cell type). However, there was no systematic report about the effect of SBT-A on A549 cells, thus, we designed the present study to investigate the antitumor effects of SBT-A on the A549 human lung carcinoma cell line.

## 2. Results and Discussion

### 2.1. SBT-A Inhibited the Proliferation of A549 Cells in Vitro

**Figure 2:**
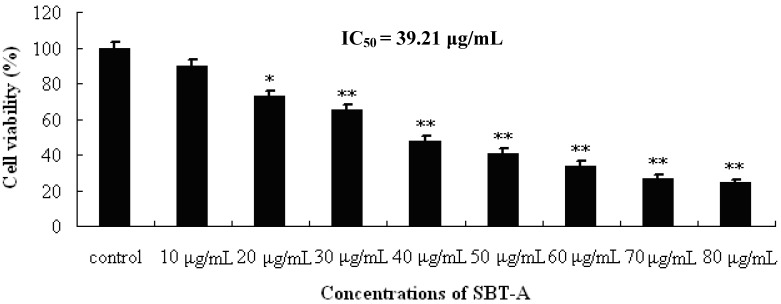
Proliferation inhibiting effects of SBT-A on A549 cells. A549 cell lines were treated with low to high concentrations of SBT-A (10, 20, 30, 40, 50, 60, 70 and 80 μg/mL) for 48 h, and cells proliferation was determined by MTT assay. The IC_50_ value was 39.21 μg/mL. The data are represented as mean ± S.D (*n* = 4). * *p* < 0.05 and ** *p* < 0.01 *vs*. control.

The antiproliferative activity of SBT-A on A549 cell line was evaluated by MTT assays. Cells were treated with a series of concentrations of SBT-A, and the cell viabilities were determined at 48 h after treatment. As can be seen from the Figure 2, our results indicated that SBT-A can significantly inhibit the proliferation of A549 cell lines at concentrations of 20 to 80 μg/mL, compared to the control group (*p* < 0.05), in a concentration-dependent manner, and the IC_50_ value was 39.21 μg/mL. The results of our present study thus revealed that SBT-A can inhibited the proliferation of A 549 cells significantly *in vitro*.

### 2.2. Effect of SBT-A on the Morphological Changes of A549 Cells

It is well known that apoptosis is a programmed and physiological mode of cell death. Cell morphological changes, extensive DNA fragmentations and apoptotic bodies are the characteristics of apoptosis, which are triggered by apoptosis signals [18].

**Figure 3:**
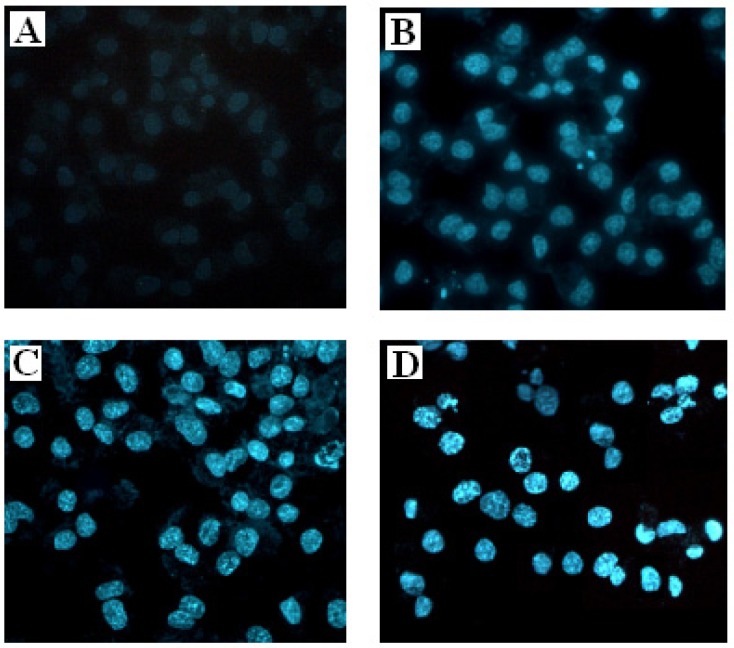
Fluorescence photomicrographs showing the effects of SBT-A on morphological changes of A549 cells by staining with Hoechst 33258 (×200). A549 cell lines were treated with low to high concentrations of SBT-A (20, 40, and 80 μg/mL) for 48 h. (**A**–**D**) represent the control, 20, 40, and 80 μg/mL groups.

To further investigate whether the antiproliferative activity of SBT-A on A549 cell lines occurs via apoptosis or necrosis, fluorescence photomicrography was applied by staining with Hoechst 33258. As shown in Figure 3, after treatment with different concentrations of SBT-A for 48 h, the characteristic features of apoptosis (including marked nuclear condensation, membrane blebbing, nuclear fragmentation and apoptotic bodies) can be seen in the A549 cells, in a concentration-dependent manner. Thus, our present results indicated that the SBT-A can suppress the proliferation of A549 cell lines though inducing apoptosis.

### 2.3. Flow Cytometry Assay of A549 Apoptosis Induced by SBT-A

In order to confirm that treatment with SBT-A can induce apoptosis, Annexin V/PI staining assay was applied.

**Figure 4:**
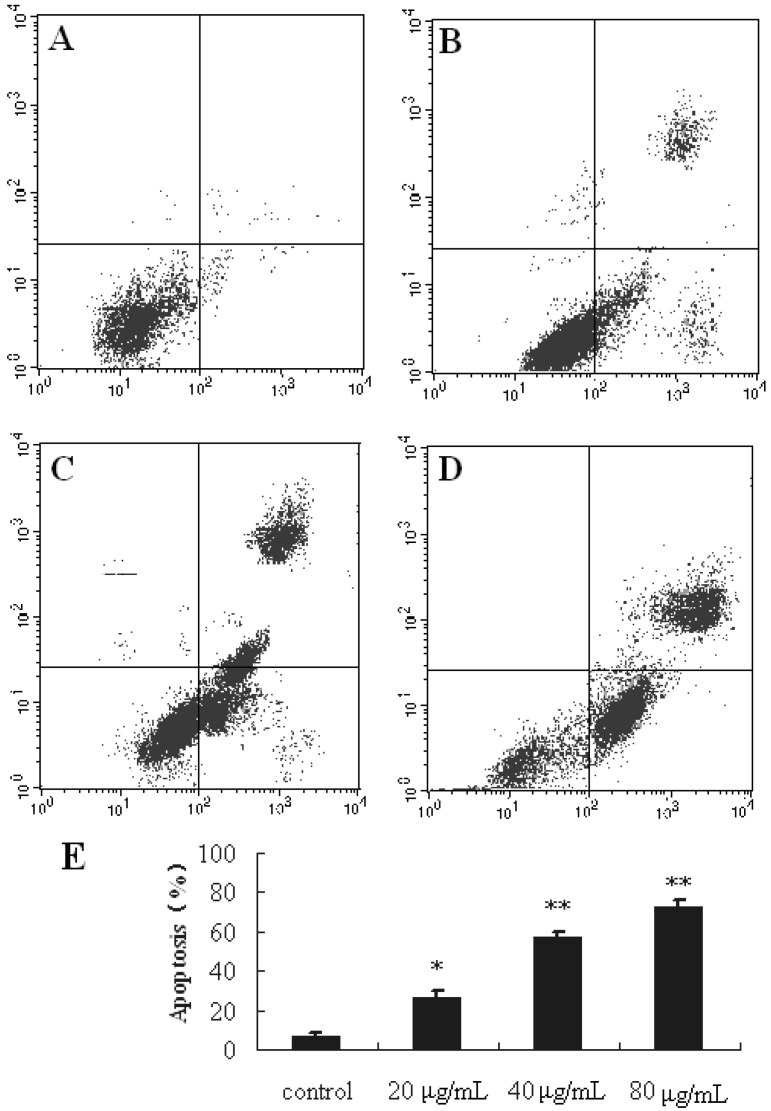
Flow cytometric analysis of A549 cells apoptosis following exposed to SBT-A. A549 cell lines were treated with low to high concentrations of SBT-A (20, 40, and 80 μg/mL) for 48 h. The flow cytometry assay was performed to determine the apoptosis rate by Annexin-V/PI double-staining. (**A**–**D**) represented the groups of control, 20, 40, and 80, (**E**) is the statistical chart of Figure 4 A–D; the data are represented as mean ± S.D (*n* = 4). * *p* < 0.05 and ** *p* < 0.01 *vs*. control.

From the results of our present study (Figure 4), after treatment with SBT-A at the concentrations of 20, 40, 80 μg/mL for 48 h, significant apoptosis can be induced (*p* < 0.05, *p* < 0.01, and *p* < 0.01 for the concentrations of 20, 40, and 80 μg/mL, respectively), in a concentration-dependent manner. These results revealed that the anticancer activity of SBT-A *in vitro* may be related to the induction of apoptosis.

### 2.4. Effect of SBT-A on the Expressions of Caspase Family and Bcl-2 Family Proteins

It is well known that killing cancer cells via apoptosis is a ideal therapeutic strategy for treating cancer [19,20]. During the apoptosis process, the mitochondria-mediated apoptosis pathway plays a pivotal role [21]. Caspase proteins are a family of cysteine proteases that can be activated during the execution phase of the apoptosis.

**Figure 5:**
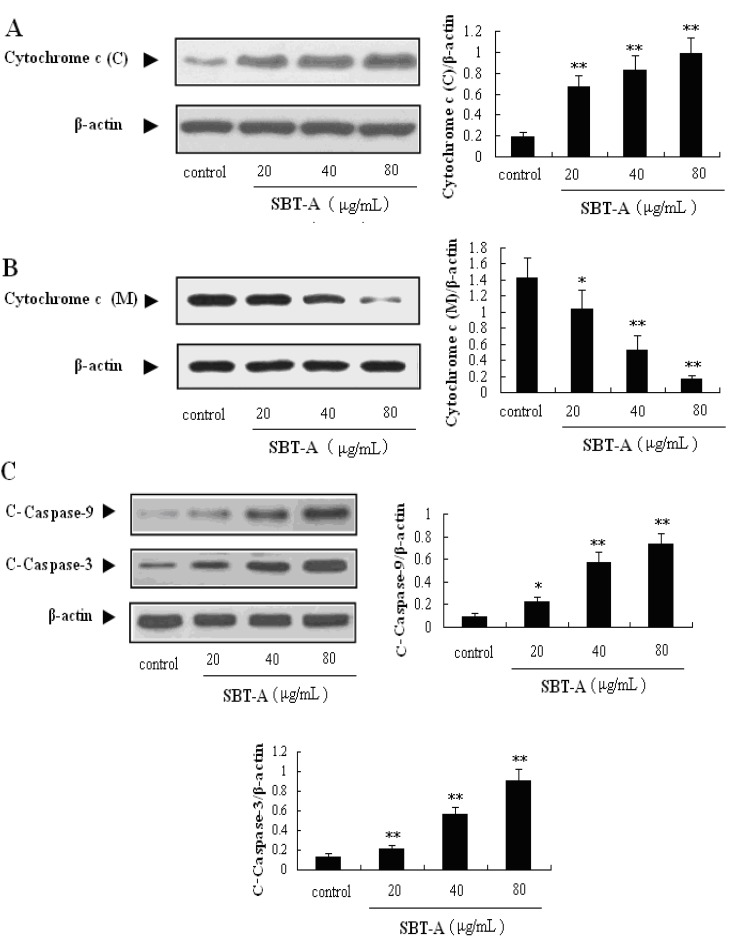
Effects of SBT-A on expressions of cytochrome c, cleaved-caspase-3 (C-capase-3), and cleaved-caspase-9 (C-caspase-9) proteins on A549 cells. Cytochrome c (C) and cytochrome c (M) mean expressions of cytochrome c in cytoplasm and mitochondria, respectively. A549 cell lines were treated with low to high concentrations of SBT-A (20, 40, and 80 μg/mL) for 48 h, then, the different proteins levels were measured by western blotting, respectively. The data are represented as mean ± S.D (*n* = 4). * *p* < 0.05 and ** *p* < 0.01 *vs*. control.

After the apoptosis is induced, mitochondria release cytochrome c in the cytosol, then caspase-9 proteins are activated by cytochrome c, commonly leading the activation of caspase-3 [22]. Then, the caspase cascades reactions can be induced after the casepse-3 was activated. Caspase-3 is one of the key proteins in the mitochondria-mediated apoptosis pathway, and is the most important executioner proteins; therefore, the activation of caspase-3 is considered to be a biochemical hallmark of apoptosis [19]. As shown in Figure 5, SBT-A can significantly promote the expression levels of cytochrome c (*p* < 0.01), caspase-3 (*p* < 0.01) and caspase-9 (*p* < 0.05) in a concentration- dependent manner.

**Figure 6:**
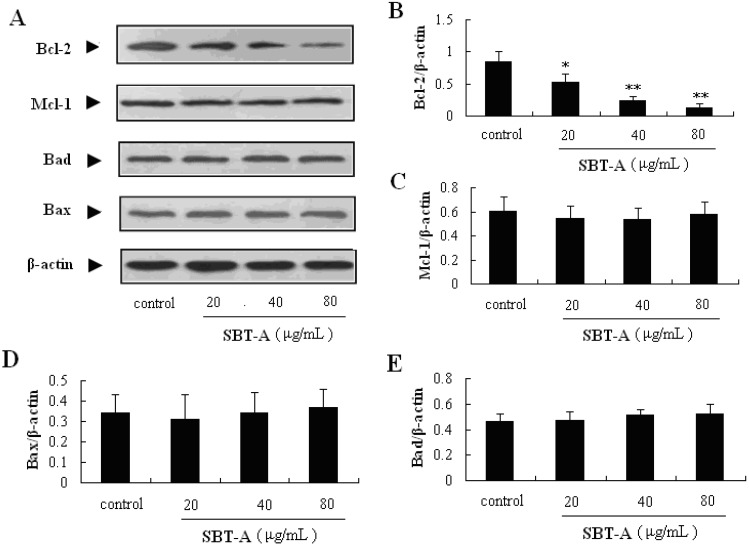
Effects of SBT-A on expressions of Bcl-2 family proteins on A549 cells. A549 cells were treated with low to high concentrations of SBT-A (20, 40, and 80 μg/mL) for 48 h, then the different proteins levels were measured by western blotting, respectively. The data are represented as mean ± S.D (*n* = 4). * *p* < 0.05 and ** *p* < 0.01 *vs.* control.

The Bcl-2 family proteins are also reported to play important role in the regulation of the mitochondria-mediated apoptosis pathway. Bax and Bad are pro-apoptotic proteins in the Bcl-2 family, whereas Bcl-2 and Mcl-1 are the well-known antiapoptotic proteins. In addition, the ratio of Bax and Bad/Bcl-2 and Mcl-1 plays a key role in the induction of apoptosis in the mitochondrial-mediated apoptosis pathway. In the results of our present study, the Bcl-2 proteins can be obviously down-regulated by treatment with SBT-A (*p* < 0.05, *p* < 0.01, *p* < 0.01), with a concentration-dependent manner (Figure 6). From the results mentioned above, we can easily conclude that SBT-A is an effective inducer of apoptosis mediated by mitochondria on A549 cell lines via the activation of caspase-3 cascade and down-regulation of Bcl-2 levels.

### 2.5. In Vivo Antitumor Efficacy Study

The results from our present *in vitro* experiments indicated that SBT-A induced cytotoxic effects on A549 cells via induction of mitochondria-mediated apoptosis. Next, we studied the antitumor effect of SBT-A *in vivo* with a transplanted tumor nude mice model. To obtain a better bioavailability, the SBT-A was administered by intraperitoneal injection. 

**Figure 7:**
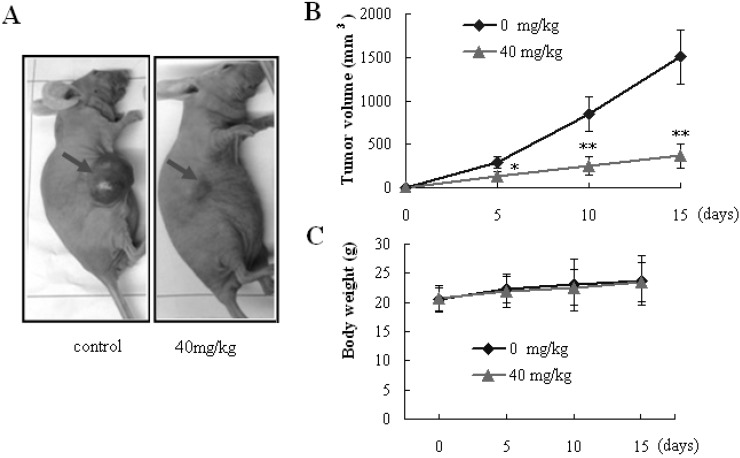
SBT-A showed antitumor activity on A549 tumor-bearing nude mice. (**A**) Illustration of a representative tumor after treatment with SBT-A; (**B**) The effect of SBT-A on tumor volumes; (**C**) The effect of SBT-A on the weights of A549 tumor-bearing nude mice. The data are represented as mean ± S.D (*n* = 10). * *p* < 0.05 and ** *p* < 0.01 *vs.* control.

**Figure 8:**
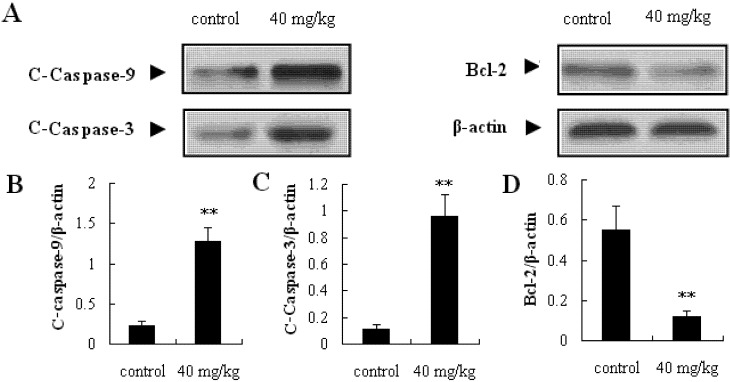
Effects of SBT-A on expressions of C-Caspase-3, C-Caspase-9 and Bcl-2 proteins in tumor tissues. The data are represented as mean ± S.D. (*n* = 10). * *p* < 0.05 and ** *p* < 0.01 *vs.* control.

Exposure of mice to SBT-A (40 mg/kg) resulted in a significant suppression of tumor growth in a 15 days’ observation period (*p* < 0.05), compared to the control group (Figure 7B). However, no obvious difference in body weight was observed between the SBT-A treatment mice and control mice (*p* > 0.05) (Figure 7C). These results from the *in vivo* investigations above showed that SBT-A had a significant antitumor effect on A549 tumor-bearing nude mice. Furthermore, caspase-3, caspase-9, and Bcl-2 proteins in the tumor tissues were determined, and as can be seen from the Figure 8, the expressions of caspase-3 and caspase-9 increased significantly in the SBT-A treated nude mice (*p* < 0.01), compared to the control mice; whereas the Bcl-2 expressions were decreased obviously (*p* < 0.01). The results of the *in vivo* experiments were also consistent with the results obtained *in vitro*, which suggested that the antitumor effects of STB-A on A549 might be related to the mitochondria-mediated apoptosis pathway.

## 3. Experimental

### 3.1. Chemicals

Scutebarbatine A was purchased from the BioBioPha Co. Ltd. (Yunnan, China); RPMI 1640 media and fetal bovine serum (FBS) were obtained from Invitrogen (Carlsbad, CA, USA). Methylthiazdyldiphenyl-tetrazolium bromide (MTT), DAPI and dimethyl sulfoxide were purchased from Sigma-Aldrich (St. Louis, MO, USA). Antibodies for caspase-3, caspase-9, Bcl-2, Mcl-1, Bax and Bad were purchased from Santa Cruz Biotechnology (Santa Cruz, CA, USA); Hoechst 33258 kit, Annexin V/PI kit was purchased from Beyotime (Jiangsu, China). All other chemicals used in this study were of analytical reagent grade.

### 3.2. Animals

Nude mice (5–6 weeks old) were purchased from the SLRC Laboratory Animal Company (Shanghai, China). All animal treatments were strictly in accordance with international ethical guidelines and the National Institutes of Health Guide concerning the Care and Use of Laboratory Animals, and the experiments were carried out with the approval of the Animal Experimentation Ethics Committee of the General Hospital of Chengdu Military Command Area.

### 3.3. Cells Culture and MTT Assay

Human lung cancer cell line A549 was purchased from Shanghai Institute of Cell Biology at the Chinese Academy of Sciences (Shanghai, China). The cells were cultured in RPMI-1640 medium supplemented with 10% fetal bovine serum and antibiotics (100 U/mL penicillin and 100 μg/mL streptomycin). The cells were subcultured till reaching logarithmic growth phase (37 °C in 5% CO_2_/95% air). Then, the MTT assay was performed to evaluate the antiproliferative effect of SBT-A on A549 cells. A549 cells (1 × 10^4^/0.2 mL) were seeded in 96-well plates and treated with SBT-A for 48 h. After that, MTT assay was carried out using standard protocol and optical density (OD) was measured at 490 nm by using an enzyme-labeling instrument (EX-800, Bio-Tek, VT, USA).

### 3.4. Apoptosis Assay by Hoechst 33258

Hoechst 33258 staining assay was performed according to a method described previously [22]. The A549 cells in logarithmic growth phase were seeded at a final concentration of 4 × 10^5^/mL in a 6-well culture plate. A549 cells were exposed to SBT-A (20, 40, and 80 μg/mL) for 48 h. Then A549 cells were stained by Hoechst 33258, and the changes in the nuclei of cells were examined and photographed by using a fluorescence microscope (Olympus, BX-60, Tokyo, Japan).

### 3.5. Determination of Cell Apoptosis by Annexin V/PI Double Staining

The A549 cells in logarithmic growth phase were seeded at a final concentration of 4 × 10^5^/mL in a 6-well culture plate. A549 cells were exposed to SBT-A (20, 40, and 80 μg/mL) for 48 h. Then A549 cells were double stained by AnnexinV/PI apoptosis detection kit following to the manufacturer’s instructions. Then the cell apoptosis was determined by using a flow cytometer (FCM, BD, NJ, USA).

### 3.6. Western Blot Analysis

Total proteins of cells or tissues were extracted, and the protein concentration was determined by using the Bradford straining method. Then, equal amounts of protein (40 μg) were separated by sodium dodecyl sulfate/polyacrylamidegel electrophoresis (SDS/PAGE), blotted on polyvinylidene difluoride (PVDF), and probed with corresponding monoclonal antibody, and subsequently with goat antirabbit/HRP, and detected by chemiluminescence. To measure protein loading, antibodies directed against β-actin were used.

### 3.7. Antitumor Activity Study of SBT-A in Vivo

Nude mice were divided into two groups (*n* = 10). All the mice were injected in the right flank subcutaneously with A549 cells (2 × 10^6^ per mouse). When the tumors grew to approximate 2–3 mm in diameter, the treatment group mice were treated with SBT-A (40 mg/kg/d, intraperitoneally injection). The control group were received an equal volume of solvent control (0.5% DMSO). All the groups were observed for 15 days, and tumor sizes were measured every 5 days. Tumor diameters were determined by using a Vernier caliper, and then the tumor volumes were calculated according to the formula [18,23]: Volume = (width^2^ × length)/2. Animals were killed immediately after 15 days of observation, and the tumors tissues were collected and homogenized for western blotting.

### 3.8. Statistical Analysis

The significance of the differences between the different groups was determined with the Student’s t-test. The results are presented as mean ± S.D, and the differences were considered significant at *p* < 0.05.

## 4. Conclusions

Collectively, our results demonstrated for the first time that the SBT-A shows significant potent growth inhibitory effects on A549 cells *in vivo* and *in vitro*, and the SBT-A can induce apoptosis by up-regulating the expressions of caspase-3 and caspase-9, and down-regulating the level of Bcl-2 in A549 cells. These findings revealed that SBT-A is worthy of development for cancer medical treatment in the future.
